# Teaching Methodologies and School Organization in Early Childhood Education and Its Association with Physical Activity

**DOI:** 10.3390/ijerph18073836

**Published:** 2021-04-06

**Authors:** Adriana Nielsen-Rodríguez, Ramón Romance, Juan Carlos Dobado-Castañeda

**Affiliations:** Human Kinetics and Body Composition Laboratory, Department of Didactics of Languages, Arts and Sports, Faculty of Educational Sciences, Universidad de Málaga, Campus de Teatinos s/n, 29010 Málaga, Spain; adriananielsen@uma.es (A.N.-R.); jcdobado@uma.es (J.C.D.-C.)

**Keywords:** children, early childhood education, physical activity, sedentary behavior, movement integration, active methodologies, accelerometry

## Abstract

Early childhood represents a crucial period for child development. Physical activity is essential in this process, but studies show that children are very inactive and do not meet the recommended minimums. Due to the large proportion of time they spend at school, it is necessary to examine active and sedentary behaviors in these environments. The aim of the study is to analyze the amount and intensity of physical activity in preschool children during the school day according to the methodology used. Using accelerometry, the amount and intensity of physical activity and sedentary behavior of 156 children aged 4–6 years at different times of the school day were evaluated. The results revealed that preschoolers spend most of their class time sedentary, with children participating in active methodologies registering the highest amount and intensity of physical activity. Recess and specific motor sessions are the most active times, although the latter should increase the time of intense activity that they imply. To increase physical activity during the school day, it is necessary to establish movement integration methodologies, while increasing the number and adjusting the duration of specific motor sessions and of recesses, so that the maximum possible use is made of them.

## 1. Introduction

Early childhood (up to five years of age) represents a crucial period for the physical, social, affective, mental and emotional development of children [1,2,3,4] in which the development of basic motor skills, perceptual-motor factors, physical capacities, and psychosocial variables should be emphasized [1,5]. Furthermore, at this robust and rapid growth stage in cognitive development, a strong connection between action and cognition begins to be built [6,7,8]. The experiences that take place during this period are the drivers of many of these changes and shape the trajectory that child development will follow [3,9,10,11]. Therefore, they must be meaningful, practical [12], integrated, and globalizing experiences, proposed from an approach based on experience, movement, child activity and play, and applied in an environment of safety, affection, and trust [1,4,13,14].

A fundamental role in this process is played by physical activity (PA), since its practice during early childhood offers a wide range of physical, physiological and psychosocial benefits [2,4,5,8], and contributes to cognitive development and learning [15,16,17]. This is because aerobic exercise can cause changes at various levels, including morphological and functional changes in the brain, and these changes have a significant effect on cognition and behavior [5,6,11,18].

There is a belief that PA is intrinsic to children’s behavior, leading to the assumption that young children are sufficiently active per se [18,19]. However, studies show that children do not meet recommended PA guidelines as early as preschool [2,18,19,20,21].

Since PA is essential to promote physical and cognitive health and well-being of young children, an accurate assessment of these factors is becoming increasingly important, with recent research highlighting the need for further evidence [1,2,19,21,22]. Nonetheless, the previously described belief, together with the challenges of measuring both PA and cognitive processes in early childhood may have limited the research carried out in this age group [8,11].

Despite the large proportion of time that the preschool-age population spends in various educational facilities, there are few studies that track PA and sedentary behavior in these settings or analyze their characteristics that correlate with active and sedentary time [2,9,23,24]. Exploring children’s behavior in these settings to obtain the necessary information that will allow us to establish appropriate interventions should be a priority [13,15,21,25].

In addition, current evidence related to active school interventions is often drawn from relatively small studies that focus on assessing the effectiveness of outcomes, rather than also analyzing the processes underlying these effects and the context in which the intervention was carried out [10,11,15,26,27,28]. Consequently, research is necessary to achieve a greater and better understanding of how specific programs and interventions can promote PA and cognitive and motor development in early childhood.

To achieve this end, the present research employs quantitative recording techniques complemented by others of a qualitative nature. Thus, the amount and intensity of PA performed by students during the school day is objectively measured using accelerometry, and this information is completed by means of a weekly schedule and a diary provided by the teachers, in which they describe the classroom routines and educational activities carried out. Thanks to this, we will be able to relate the PA performed to a specific context (in this case, the different teaching methodologies, recesses, specific motor sessions or free time after lunch), making it possible to program these moments according to the needs and possibilities of each particular center.

### 1.1. Importance of Physical Activity in Physical and Cognitive Development and Academic Performance

Brain functions and mental processes depend on an motor component existing in every cognitive act that is essential for early childhood development [18,29], and the beneficial effects of PA, especially at moderate to vigorous intensities (MVPA) and that require the participation of higher order cognitive processes, on certain aspects of brain function particularly important for education have been demonstrated [11,15]. Among other benefits, PA causes different permanent changes at the structural and functional level in various brain areas [10], involving systems related to its maintenance and plasticity and, therefore, in learning [10,29,30]. This impact of PA on cognitive function occurs through several processes. First, there is an increase in cerebral blood flow that leads to an increase in oxygenation and glucose supply to certain neural areas closely related to cognitive functions of educational relevance [10,11,17,30]. In parallel, modifications of white matter integrity and gray matter volume can be observed, partly produced by neuronal maintenance and proliferation, leading to memory consolidation and improved cognitive function in children [5,11,15].

In addition, there is a documented effect on the proper balance of neurochemicals necessary for learning. After the practice of PA, the levels of brain neurotrophic factors such as brain-derived neurotrophic factor (BDNF), insulin-like growth factor 1 (IGF-1) and vascular endothelial growth factor (VEGF) are regulated (and even increased), which has been associated with improved short- and long-term learning [10,17,30]. In short, experimental research in preschool-age children indicates that MVPA is associated with improved higher-order cognition and adaptive and goal-directed behavior (executive functions) [3,18], academic performance, on-task time engagement, desirable classroom behavior [4,11,26] and, in general, with proper overall development and improved health status [4,16,19,31,32].

What is more, there is evidence that not all forms of aerobic exercise benefit the executive function equally, being cognitive-engagement exercise the one that has a stronger effect on it [6,15,17]. For example, problem solving that occurs during motor play may promote higher-level skill such as attention-inhibition, working memory, and cognitive flexibility [3,5,6], in addition to other cognitive functions such as planning, organization, sequencing, and decision-making [12]. These cognitive skills are believed to be linked by some common underlying processes, but they are employed and combined differently depending on the task to guide behavior, being necessary not only for academic success, but also for success in daily-life tasks that all children must master to gain full independence [3,6,17,33].

Thus, regular PA and movement integration programs have a great impact on aspects such as cognitive performance [6], response speed, motivation, working memory, planning, attention, inhibitory control [5,7,11,15,17,26,27], social and emotional development and, in general, executive functions and academic performance [3,4,18,19].

In this way, we can state that there are at least three pathways through which exercise can facilitate cognitive functioning: the short- and long-term physiological changes in the brain induced by aerobic exercise, the cognitive demands inherent in engaging and goal-directed exercise, and the cognitive engagement required to execute and coordinate complex motor movements [6,11,17].

### 1.2. Physical Activity and Sedentary Behavior. Role of Schools and Recommendations of Official Organizations

In recent years, researchers have begun to explore sedentary time as a unique construct, rather than simply the opposite of PA [2,9,21], since several studies have repeatedly shown that children spend most of their free time in sedentary activities [5,16,21]. At the same time, a large number of short- and long-term health consequences, associated with low levels of PA and excessive time of sedentary behaviors during the first five years of life have been documented [2,9,20,21,23,28,34]. In response, the promotion of PA has become a priority for health agencies, and several official organizations have developed PA and sedentary behavior guidelines for young children aged 0–5 years [9,34].

Faced with the alert of the worldwide physical inactivity of this population sector, the World Health Organization has issued for the first time in its history a report with global recommendations and general activity standards in children from 0–5 years old. In this document, together with those published by other organizations in the same line, it is pointed out that, although all PA carried out is beneficial, children under five years of age should perform a minimum of 180 min of PA per day of various types and of any intensity. Of this time, at least 60 min will be preferably of aerobic MVPA [35,36,37,38], recommending that they do not remain seated for a long time [37,39].

Regarding what type of activities and what durability are necessary to represent a significant factor and obtain the best possible results, it is recommended that this MVPA consist of the practice of games, sports, displacements, recreational activities, physical education or programmed and structured exercises. This type of PA, performed assiduously and repetitively, causes the aforementioned alterations, and can become long-lasting [1,5,17,37]. However, despite the many benefits of physical exercise during early childhood, currently PA levels in preschool-age children are very low and very few children meet the minimum daily recommendations [2,5,16,18,20,21,26,31].

For this reason, studies like this are necessary to analyze PA and sedentary behaviour in specific circumstances and contexts (such as schools), taking into account their underlying elements (e.g., teaching methodologies or school organization), in order to be able to implement actions to improve the current situation. In this sense, the main contribution of this work is the use of more precise and objective measures of PA through accelerometry at different types of schools and environments, to see if their differences have any association with different levels of PA, as well as its ability to detect effects at different levels in school systems.

Added to this, there are difficulties in increasing levels of motor practice in childhood because the nature of children’s play has changed in recent generations and they no longer play as they did before [8], so that new technologies prevail over the performance of motor activities [19,22]. In fact, several studies have shown that children spend most of their free time in sedentary activities, spending between 39.49 min/h and 40.64 min/h in sedentary behaviors [5,16,21]. Thus, for many children, structured movement programs may be the only opportunity for PA during the day [8].

For its part, PA in schools has steadily declined in recent decades, so that currently between 73% and 89% of the time children spend in it is sedentary [7,18,21,27,40]. Furthermore, schools are finding it increasingly difficult to allocate time for PA during the school day, as there is a tendency to increase the time for subjects such as language, mathematics or science [10,12,20,41]. As a result, physical education classes, recess, and other times conducive to PA are often reduced or eliminated [20,31]. However, schools are an ideal natural environment to promote PA from an early age [16,19,27,31,42], as children spend a large number of hours per day there, have availability of resources, and movement integration interventions into the school schedule in the early years are more likely to be successful by ensuring access to the majority of the child population for extended and regular periods of time [2,20,26,27,40,41,42].

For this reason, identifying opportunities for PA at school is imperative to promote movement in this population [42]. For example, the regular classroom may be the ideal setting to combine PA with content learning if teachers implement physically active academic lessons, which would benefit students’ health, cognitive function and academic performance while preserving the time allocated to the teaching and learning process [7,20,27,28,40].

### 1.3. Active Methodologies and Movement Integration in Early Childhood Education

One strategy to increase PA during the school day is through movement integration programs, which are defined as the introduction of PA of any level of intensity during normal class time, usually as a means of teaching academic content [4,42] or as active breaks [41]. This strategy should be implemented as part of a comprehensive PA program in which movement opportunities are provided during children’s time at school [41]. However, few studies focus on classroom interventions in which PA has been integrated into academic content, despite these interventions improving students’ PA levels during school hours without affecting academic teaching time [16,20,42].

In addition, movement integration programs provide a rich pedagogical environment that improves concentration, engagement, classroom behavior, motivation, and PA among children, which can support later learning and facilitate better academic performance [4,10,11,42]. Added to this, the different types of PA that can be performed during the school day (integrated PA, recess and free play, and motor skills sessions) bring into play different types of tasks that involve executive functions, since success in these tasks depends on a goal-directed behavior that adapts to new parameters each time, rather than automatic behavior caused by associative learning [1,3,6].

The coordinative and cognitive complexity involved in movement-based tasks is seen as a mechanism through which PA characterized by novelty, diversification, complex, controlled and adaptive movements, as well as a cognitively demanding design, impacts on executive function beyond the more commonly studied role of exercise-related metabolic and physiological changes [11,15,17,18]. A fundamental resource for movement integration into academic content is play, as it involves an activity in which children engage for personal enjoyment, at the same time that they are intrinsically motivated to face different challenges and optimize their own brain development [4,25,33]. The nature of play depends to some extent on child’s physical, intellectual, and social development, but at the same time, play itself helps to develop these areas. Therefore, if PA is to be understood, assessed, and promoted in young children, this should occur in the context of active play or motor play [1,15,25].

However, PA levels in schools depend on the space available, the methodology used, as well as the presence or absence of specific motor sessions and recess [19,21,31]. For a movement integration proposal to be effective and to last over time, it is necessary to know and understand the associated factors that will affect its implementation [8,13,16,17,21,27,28,42]. First, it is important to have the support of a school principal that recognizes the importance of introducing movement from early childhood and who promotes communication among teachers to achieve greater consistency in practice [16,27,28,41]. Also, teacher training, perceptions and characteristics are important, so that movement integration is affected by individual teaching styles, teacher confidence in movement integration, the importance they bring to PA, and their ability to have a flexible approach to teaching academic content [13,16,27,28,41,42].

On the other hand, the characteristics and limitations of the school environment must be taken into account. There are aspects such as the physical space available in the center and in the classroom, the climate and its impact on the opportunity for outdoor movement integration [8,13,16,21,28,41,42] or the existence of school uniforms, which may interfere with the feasibility of movement integration [27]. It is also necessary to take into account the students in order to achieve movement integration, including their individual differences, physical and cognitive abilities, developmental stage, and individual/group preferences [11,27,42].

Finally, time constraints emerge as a key factor influencing the implementation of movement integration initiatives. Teachers have limited time for planning, programming and implementing movement integration interventions, which must also be adjusted and meet the curricular objectives for to the stage. Therefore, in most cases it is time constraints, together with teacher’s work overload, that seem to hold back the promotion of PA at school [27,28,41,42].

All these factors and variables will be taken into account in the methodology, and they will be discussed in greater depth both in the discussion and in the limitations of the study (in the case of those that finally could not be considered for some reason).

#### 1.3.1. Integrated Physical Activity (Physical Activity into the Classroom)

Active learning through integrated PA in the classroom consists of the use of movement as a means of teaching and learning academic content [40,41]. This activity, linked to an established curriculum [7], not only increases the amount of PA carried out by students, but also benefits their academic performance and their ability to remain attentive and engaged with the task [4,26].

There are several methods that can be used to integrate PA into the classroom, known as movement integration interventions. In general, these strategies involve the infusion of PA into regular class time using activities specifically designed to promote learning through movement, but they can also consist of a complete environmental restructuring as well as the introduction of equipment and activities in the classroom to reduce sedentary time [27,41].

In any of its variants, these interventions are relatively easy to program and implement in the classroom [27,42,43], being able to considerably reduce sedentary time while presenting numerous additional academic benefits. These include improved on-task behavior, greater motivation and enjoyment of learning [20,27,31,42], development of “learning to learn” by placing children in novel, cognitively challenging and complex situations that train a controlled and adaptive thinking based on cognitive flexibility [15,18], building meaningful and relevant learning [1,6], and increasing emotional self-regulation necessary for school readiness and success [11]. Thus, preschool interventions that create student-centered and action-based classroom environments impact more positively on executive functions compared to more traditional, teacher-centered classroom settings [6,22].

#### 1.3.2. Specific Motor Sessions

Although in this work we will use the concept of “specific motor sessions”, we should know that, when we talk about Physical Education in preschool, different authors use a large number of terms such as psychomotor education, psychomotor skills, physical education of base, body expression, kinesiology education or pre-sport education [1].

Specific motor sessions in early childhood are defined as a curricular area offered in schools that enhance children overall development [4,44]. This is because it enables students to develop interconnected motor and cognitive skills, understand movement concepts, participate in regular PA, maintain healthy levels of physical fitness, develop responsible personal and social behavior, and value PA, adopting healthy and physically active lifestyles [1,32,43]. In addition, they reflect an integration of dynamic systems, including musculoskeletal, cardiopulmonary, and neurological body systems; sensory systems, central sensorimotor integrative mechanisms, and motivation [4,5,8,18,25].

Specific motor sessions should be deliberate, so they are planned and designed for a specific purpose, require coordinated cognitive and physical effort, and are relevant to promote skill development [4,8]. For this reason, a PA intervention enriched with cognitive and coordinative challenges integrated into playful activities through body expression, motor, rhythm and spatial structuring games [32] seems to be the most appropriate framework to improve cognition in children by offering a greater number of benefits and positive outcomes [17,18].

A single 30-min, adult-led period of structured motor activities involving basic motor skills has been found to increase preschool-aged children’s performance in cognitive control and academic achievement [15], while improving their motor development and social skills [4,44]. However, despite its importance, studies reveal that in most centers only one session is held per week, very few carried out two or more, and some do not provided any session at all [1,44]. This may be due to the lack of specific spaces for this purpose (30% are carried out in inappropriate spaces such as the classroom itself, corridors, playgrounds, etc.), the excessive number of students, the shortage of time and material resources, the lack of specific teacher training, and the little value that the early childhood educational curriculum gives to motor work [1,4,11,13,21,32,44].

#### 1.3.3. Recess and Free Play

Free play is defined as a form of gross motor movement in which young children consume energy during PA, whether structured or unstructured, that are intrinsically motivating, provide immediate gratification, and are specifically designed to maximize enjoyment [4,8]. Recess is the time of the school day par excellence that provides children with the opportunity to engage in this type of play [43], although there are other moments when more or less free play activities are organized by school, but that occur outside school hours, such as the time before and after lunch.

Studies suggest that free play may be a potentially useful approach to increasing the amount of PA children engage in, while also generating additional benefits [8]. Although the practice of PA has documented benefits such as those already mentioned above, play is also related to development and learning, due to its impact on brain functioning [11]. Immediately after play, brain activity is modified, allowing children to perform better with regard to cognitive control, attention focus and impulses inhibition when performing a task, which can improve thinking and learning, turning moments of recess and free play into valuable supports for learning [11,15].

In addition, free play allows children to set their own rules, make choices, determine the start and end time, enhance their creativity, control their actions, solve problems, ask and answer questions, get along with their peers, and be emotionally resilient. However, this type of play is in decline and the school needs to promote it [1,4].

However, there is evidence to suggest that structured free play is more efficient than unstructured free play in producing high motor engagement [14]. Studies found that a totally free play session, unlike a structured session, ends up promoting sedentary behaviors, while structured free-play programs are more effective in improving and maintaining motor skills [8] and significantly increasing MVPA in children [14].

In summary, and in view of the above, given the need for more evidence regarding PA and sedentary time in preschool children, exploring these behaviors in the educational setting should be a priority.

That is why the aim of this study is (1) to analyze the amount, intensity levels and patterns of PA and sedentary behavior in Early Childhood Education children throughout the school day and in its different moments depending on the teaching methodology employed in class, the specific motor sessions and recesses, (2) determining their contribution to compliance with the recommendations established for this age group, and (3) making these data available to the educational community to provide an incentive for the dissemination and implementation of more active pedagogies by teachers, helping to guide the design of future school interventions.

Furthermore, with respect to the amount and intensity of PA performed, there may be variations depending on a number of factors such as the established schedule, the configuration of the classroom environment, teaching practices, and the deliberate/free play opportunities offered throughout the school day, among others. Thus, our research hypotheses are that (1) the use of active methodologies can increase the amount and intensity of PA performed by students during the school day and reduce sedentary behaviour; (2) increasing the number of specific motor sessions during school hours can decrease sedentarism and increase the amount and intensity of PA performed by students; and (3) the increase of the amount of recess time and free play moments during school hours implies an increase in the amount and intensity of PA performed by children, minimizing sedentary behaviors.

## 2. Materials and Methods

### 2.1. Study Design

A quantitative study was carried out in this research, using an ex post facto, descriptive, comparative and cross-sectional approach.

The amount and intensity of PA performed by different groups and at different educational moments (class methodology, specific motor sessions and recess and free play) were measured. Data obtained in each group were described, comparing then the PA between the groups according to the different educational moments.

### 2.2. Recruitment, Participants, Ethics Approval and Consent to Participate

For this study, centers in which the second cycle of Early Childhood Education (3 to 6 years) is taught were selected, due to their different methodologies. Within this framework, schools and classes could be recruited through intentional or convenience non-probability sampling, but not specific individuals. Therefore, the design of the present research was a randomized cluster design, with children in the same school and class sharing the same environment.

The criteria selection responded to the need to analyze centers with clearly differentiated methodologies or school organizations that could influence the PA performed by students, trying to ensure that the total number of records per center was balanced. The final sample consisted of those students who provided an informed consent signed by their parents or guardians and whose PA records met the requirements as explained in the ‘Procedure and Data Collection’ and ‘Data Management’ sections.

Finally, the sample consisted of a total of 156 children (73 girls and 83 boys) aged between 4 and 6 years (M = 5.2; SD = ±0.8) belonging to 7 classes from 4 different schools. The study was approved by the Ethical Committee of Experimentation with human beings of the University of Malaga (114-2020-H) and was conducted under the Declaration of Helsinki [45]. In addition, all procedures were reviewed and approved by the school principal of each of the participating centers, who provided the corresponding written collaboration agreements, as well as by the families. Written informed consents were obtained from the parents (or guardians) before the children joined the study. All parents received a document specifying the goals, limits and risks of the study, the methods used and the activities to be performed before being asked to sign the consents.

In any case, participation in the study was always voluntary and confidentiality regarding the identity of the participants was always guaranteed. To this end, all personal data collected were anonymized using a unique identification number, with all printed data securely stored in locked filing cabinets and electronic information stored on password-protected university computers/servers.

### 2.3. Measures and Instruments

In the present study, the amount and intensity of PA carried out, as well as the time spent in sedentary behaviors, by children between 4 and 6 years of age at different times of the school day were measured and evaluated. Following previous research, PA integrated into classroom content, recess and free play periods, and specific motor sessions were considered as times that promote PA in schools [1,3,6,20,31].

For this research, as detailed in the previous section, we understand integrated PA (or movement integration interventions) as these strategies that use movement into regular class time to teach and learn academic content [40,41], including environmental restructuring as well as the introduction of equipment and activities in the classroom to reduce sedentary time [27,41]. Additionally, when talking about specific motor sessions, we refer to scheduled, planned and designed for a specific purpose sessions that require coordinated cognitive and physical effort, and are relevant to promote overall development [4,8,44]. Finally, we consider recess and free play as those moments in which children consume energy during the practice of PA based on gross motor movement, whether structured or unstructured [4,8]. 

To obtain the most accurate measurement of PA levels and sedentary lifestyles at early ages, the nature of childhood movement patterns should be considered when selecting an instrument [19,23]. This is because PA in young children is characterized by brief bouts of intense activity interspersed with frequent rest periods [5,14,23,25,33,46], so the instrument used must be sensitive enough to detect and record sporadic and intermittent activity.

Although different methods and instruments have been described to measure PA and sedentary behaviors in children [19,24], in this research we have used triaxial accelerometry as a widely recognized method for this purpose, using the ActiGraph wGT3X-BT^®^ accelerometer (Actigraph, Pensacola, FL, USA). It is considered the most valid and reliable [21,23,24,40], has good psychometric properties compared to other types of accelerometers used in children [20], and has been used previously in similar studies carried out in the same age group [8,20,21,23,24,40,46,47].

ActiGraph wGT3X-BT^®^ accelerometers record movement in three orthogonal axes: vertical (Y), horizontal (X) and anteroposterior (Z). Its dimensions are 4.6 × 3.3 × 1.5 cm; their weigh is 19 g and have up to 16 MB of memory [23]. In order to accurately capture the variability of infant activity and retain the maximum amount of data possible, it is recommended to use the shortest possible epochs [46], so we set epochs at 1-s intervals.

Finally, after analyzing the available studies with children in preschool education [8,24], we selected the cut-off points of Pate, et al. [48] to classify PA as sedentary (0–799 counts·min^−1^), light (800–1679 counts·min^−1^), moderate (1680–3367 counts·min^−1^) or vigorous (≥3368 counts·min^−1^).

### 2.4. Procedure and Data Collection

Once the pertinent written informed consent had been obtained from the centers and families, we proceeded to the objective evaluation of the amount and intensity of PA of the children by using triaxial accelerometry. This process was developed following the indications and recommendations formulated in previous studies and research, with similar characteristics and carried out in this same age group [8,20,21,23,24,40,46,47].

The accelerometers were charged and programmed to start measuring at 00:00 h on the day they were placed, and the end of the recording was set at 23:59 h on the last day of recording. For the initialization of the accelerometers, the ActiLife^®^ software (version 6.13.3) from ActiGraph was used, as well as to later download the data.

Although they can be affixed to different parts of the body [46], in this research the accelerometers were placed above the iliac crest of the right hip of each participant with an elastic belt within 10 min prior to the onset of the school day and were removed within 10 min after the dismissal [20,40].

Data collection was carried out in 4 schools for 5 consecutive days, and PA was measured only during in-school period, which meets the minimum recommendations for reliability [8,20,21,23,24,40,46]. Five hours were recorded daily, except in one of the centers that uses split morning and afternoon day and extended the daily school day by one hour. The daily record was 300 min/day, except for the center that extends the school day, in which the daily record was 360 min/day. Children who had valid accelerometer data for at least 3 days were included in the analysis [23,46].

The researchers distributed and placed the accelerometers to the participants the first time, taking advantage of this moment to provide both teachers and children with the necessary instructions on how to use them. Likewise, they collected them once the week of data collection was over after the recording period. At that time, the teachers provided a weekly schedule and a diary in which they described the classroom routines and the teaching activity carried out in order to be able to relate them to the accelerometry data.

As the accumulated counts can be separated by time to obtain the data in one-hour intervals, together with the analysis of the schedules and diary entries, allowed us to classify the data in one-hour intervals for calculation of in-class PA depending on the different teaching methodologies, recess, specific motor sessions or free time after lunch. This type of classification has been successfully carried out in other similar studies [40].

Recess was considered as one-hour interval in which children had breakfast and free time for play, usually outdoors. Motor classes or sessions were defined as one-hour time intervals that included the displacement to and from the space where the session takes place. Free time after lunch was estimated as the one-hour period in which the children had lunch and then had the rest of the time for free play before returning to class in the afternoon.

With regard to PA in the classroom, each of the centers has a different school organization and teaching methodology that directly affects the movement performed by the children. Center 1 has a continuous 5-h school day. The day includes a 60-min recess for breakfast and free play for the children. This center implements an active methodology for one hour per day based on small learning stations called learning corners, located inside the classroom and in a small space attached to it for the exclusive use of that group of students, and among which children can move freely according to their interests. There is only one specific motor session weekly.

The school day at Center 2 is divided in two parts, 4 h in the morning and 2 h more in the afternoon. The morning day includes a 60-min recess for breakfast and free play. After the morning session, some children stay at school for lunch and continue with the additional 2 h in the afternoon. The rest of the children go home for lunch and return later to continue the school day. In this intermediate period, children who stay in the center have 60 min to have lunch, and then play freely. This center employs a traditional methodology in which the student spends most of the time passively seated, although small doses of movement are sometimes provided for specific activities. This center encourages the practice of PA by including in its schedule 3 specific motor sessions per week. Center 3 has a continuous 5-h school day, including a 60-min recess for breakfast and free play. This center also employs a traditional methodology based basically on passive attention of the child from his chair, although it tries to increase the practice of PA by including in its weekly schedule 5 specific motor sessions, at a rate of one 60-min session per day.

Finally, Center 4 is also organized in a 5-h continuous school day and, in the same way as in the previous cases, has a 60-min recess used for breakfast and free play. However, this center implements a methodology of open concept classroom, based on the possibility of sharing spaces (classrooms, corridors and outdoor spaces), which have been previously prepared by the teachers to favor different teaching and learning experiences. In this way, each classroom and space is arranged according to a series of curricular contents and the students can move freely among them, going from one classroom to another and among the different spaces of the center. Meanwhile, they are guided, accompanied and advised by the teachers, who also record the children’s use of each space and take evidence that allows them to evaluate the evolution of the students’ learning while checking the effectiveness and acceptance of each of the educational proposals offered. In this case, the weekly schedule does not include any specific motor session, but there are spaces provided for this purpose in which the students can play, exercise or move freely.

### 2.5. Data Management 

The accelerometer data were downloaded and processed using ActiLife^®^ (Actigraph, Pensacola, FL, USA) software (version 6.13.3) on the same computer on which they were initialized to avoid differences due to possible desynchronization between computers. The cut-off points of Pate et al. [48] were used, as in studies with similar ages [8,24], and moderate and vigorous intensity PA were combined to determine the time spent in MVPA, which would be consistent with public health recommendations for PA in children [46]. Once the accelerometer data were downloaded and processed, Microsoft Excel^®^ was used to prepare and clean them. Data from the class diaries/schedules were also manually transcribed into a Microsoft Excel^®^ file. Similar data processing criterion from similar studies was used [20,21,46]. Only the physical activity levels of the children while they were in the school were taken into account, obtaining the total valid hours per school day and the total time spent in sedentary behavior, light, moderate, vigorous and MVPA. Wear time calculation does not exclude all zero counts from the data, as children may engage in prolonged periods of sedentary behavior in the classroom, and this is part of the data.

Based on existing precedents on the use of accelerometry data, a day was considered with complete information if the sensor was worn for at least 3 h (≥3 h of wear time) on the same day, and only children with 3 valid days or more were included in the study [23,46]. The accelerometer was considered as not worn if it was for a 24-h period recording zero consecutive counts. The second step in data reduction involved eliminating participants with an insufficient number of days with complete data. This elimination only affected weekly PA data, and single-day data could be used for other types of fractional PA analyses in which activity and sedentarism data were evaluated during different moments of the day (teaching content using different methodologies, motor sessions, recess and free play, etc.). All PA levels were averaged to minutes/hour or minutes/day, in order to facilitate comparison between different participants and centers.

### 2.6. Statistical Analysis

The refined data were then transferred for analysis to the IBM SPSS^®^ 24.0 statistical package (IBM Corp, Armonk, NY, USA) in its Windows^®^ version.

Once all the data were prepared and classified, a descriptive and inferential analysis was carried out, and the results were expressed as percentages, means and standard deviations. The level of significance was set at *p* ≤ 0.05 for the different tests. Normality tests revealed a non-normal distribution, so nonparametric tests were used for comparisons between groups. First, the Kruskal–Wallis test was used to check whether the compared groups were significantly different. Then, the Mann–Whitney U test was used, applying the Bonferroni correction, to analyze the differences by pairs in terms of PA and sedentary behavior developed in each center (1) during the school day, (2) according to the teaching methodology used, and (3) in the specific motor skills sessions, recesses, and free play times.

## 3. Results

Data analysis revealed that children in Early Childhood Education spend most of their class time sedentary. This is evident in Table 1, which shows in minutes the descriptive values in the form of the mean and standard deviation referring to the different levels of PA carried out in each school, as well as the percentage of PA carried out according to the total number of minutes of the school day. This information is completed with the data from Table 2, which shows the existing difference in relation to the PA carried out according to the school day in each center.

When examining both tables, we observe that Center 4, which employs an open concept classroom methodology with shared spaces, has the lowest levels of sedentary behavior in absolute terms (237.5 ± 11.0 min/day out of 300 total), although this number is still very high (79.1% of the day). However, this difference in student sedentary behavior is not so significant compared to Center 3, which implements a specific daily motor session (Z = −1.008; *p* = 0.313). Center 4 is also the one with the highest level of light PA (20.7 ± 2.6 min/school day), not being this difference significant (but almost) compared to Center 2 (Z = −2.420; *p* = 0.016). It should be recalled that this center does not dedicate any specific session to motor skills but offers the children spaces for motor practice in which they can play while exercising.

In relative terms, Center 1, which employs an active methodology for 1 h per day and only one specific motor session is taught, is the center that achieves the worst results (82.4% of the school day). This difference is significant with Center 2 (Z = −7.734; *p* = 0.000), Center 3 (Z = −2.874; *p* = 0.004) and Center 4 (Z = −3.205; *p* = 0.000). Center 2, which has a longer school day (360 min) and three specific motor sessions per week, is the center that reaches the best results in relative terms (77.7% of the school day). However, if we talk about absolute terms, we find that Center 2 has the highest sedentary time (280.0 ± 11.5 min), with a significant difference compared to Center 1 (Z = −7.734; *p* = 0.000), center C (Z = −7.898; *p* = 0.000) and Center 4 (Z = −6.623; *p* = 0.000).

With regard to MVPA, the center with the best results in absolute terms is Center 2 (44.6 ± 8.1 min/day of 360 h), in which 3 weekly motor sessions are taught. This difference does not become significant compared to Center 3, which implements one daily motor session (41.9 ± 9.6 min/day of 300 h) (Z = −1.904; *p* = 0.057). In relative terms, the greatest amount of MVPA is performed by children in Center 3 (14.0% of the day) and 4 (13.9% of the day) respectively. Let us remember that Center 3 offers is a daily motor session and that Center 4 uses an open concept classroom methodology without any specific motor session. The values reached by these centers are very similar in this regard, both in absolute terms (41.9 ± 9.6 min vs. 41.8 ± 9.0 min; *p* = 0.313) and relative terms (14.0% vs. 13.9%). Again, Center 1 is the one that achieves the worst results both in absolute (34.9 ± 8.9 min) and in relative terms (11.6% of the day).

Regarding LVPA or total PA, in absolute terms it is Center 2 that reaches the highest levels of PA (63.5 ± 10.3 min/day), closely followed by Center 4 (62.5 ± 11.0 min/day). We have to take into account here that the school day for Center 2 is 360 min, while the school day in Center 4 is 300 min. However, there is no significant difference between Center 2 and Center 3 (Z = −1.878; *p* = 0.060) or with Center 4 (Z = −0.467; *p* = 0.640). In relative terms, it is Center 4 with open classrooms, in which access to outdoor spaces is allowed, the one that reaches the best results (20.8%). However, this difference is only significant with Center 1 (Z = −3.205; *p* = 0.001), which is the one that reaches the lowest levels of PA both in total and in most of each of the intensity levels.

On the other hand, Table 3 shows the descriptive values in form of the mean, standard deviation and percentage of the different levels of PA performed by children, expressed in minutes per hour, depending on the teaching methodology or organization used in the classroom, while Table 4 shows the differences in the records of each level of PA according to the methodology or organization used.

In relation to our first hypothesis, which refers to the relationship between PA and the methodology used, the results showed significant differences in each of the comparisons made between the different levels of PA required by the different methodologies (Table 4). Considering the values recorded in Table 3, we can report that the traditional methodology shows the worst results in terms of sedentary behavior, both in absolute and relative terms (55.4 ± 2.4 min/h; 92.3%), as well as the worst results in terms of PA performed for each level of intensity (light = 1.9 ± 0.9 min/h; 3.1%. Moderate = 1.7 ± 0.9 min/h; 2.8%. Vigorous = 1.1 ± 0.7 min/h; 1.8%. MVPA = 2.7 ± 1.5 min/h; 4.5%. LVPA = 4.6 ± 2.4 min/h; 7.6%).

The methodology based on learning corners reached intermediate values between the traditional methodology and the open classroom concept one, both in sedentary behavior (52.5 ± 3.3 min/h; 87.5%) and in the different levels of intensity of PA (light = 2.7 ± 1.0 min/h; 4.5%. Moderate = 2.5 ± 1.2 min/h; 4.2%. Vigorous = 2.2 ± 1.4 min/h; 3.7%. MVPA = 4.7 ± 2.4 min/h; 7.8%. LVPA = 7.4 ± 3.3 min/h; 12.3%), while in the one based on open classrooms, the best records were reached in both sedentary behavior (46.6 ± 5.9 min/h; 77.6%) and in all levels of PA intensity (light = 4.5 ± 1.5 min/h; 7.5%. Moderate = 4.6 ± 1.9 min/h; 7.6%. Vigorous = 4.1 ± 2.6 min/h; 6.8%. AFMV = 8.7 ± 4.3 min/h; 14.5%. AFLV = 13.1 ± 5.9 min/h; 21.8%), despite not dedicating any moment to specific motor sessions. According to these results, we can say that active methodologies can increase the amount and intensity of PA performed by students during the school day and reduce sedentary behavior, thus confirming our first hypothesis.

Another element to take into account when estimating the time and intensity of PA during the school day is the number and content of specific motor sessions. The registered centers include in their weekly schedule a different number of them. Center 1 contemplates 1 weekly session, Center 2 implements 3 sessions, and Center 3 includes 5 sessions. Center 4 does not carry out any session, but has a space specially designed for this purpose. Table 5 shows the mean values and the standard deviation of recorded PA intensities in the specific motor sessions of our sample (SS = 379).

Regarding the effectiveness of specific motor sessions to increase the amount and intensity of PA performed by students during the school day, it is possible to say that our second hypothesis is not fully achieved. This is because we must take into account that the content of these sessions, the space in which they are held, the materials and the methodologies employed, and the type of PA implemented will be decisive. For this reason, although Center 3 dedicates an hour daily to motor activities, its overall PA records are not much higher than those of the other centers. We can observe that, out of a planned duration of 60 min, the mean total time of PA is 17.2 ± 5.2 min/h, occupying sedentary behavior 42.8 ± 5.2 min/h (Table 5). The mean values of MVPA were 12.6 ± 4.5 min/h (Table 5).

Recesses and the activities that students execute during them also have a great influence on the PA that children perform during the school day. The centers analyzed in our study only contemplate a 60-min recess. During this time, children have breakfast and then can play freely in the playground. As shown in Table 6, in the studied centers children present a mean sedentary behavior of 39.3 ± 6.3 min during the 60-min recess, while MVPA reached mean levels of 14.9 ± 5.5 min and total physical activity was 20.7 ± 6.3 min out of the total 60.

During these minutes in which children can play freely, we can appreciate how, according to the selected cut-off points, their movement tends to be closer to moderate and vigorous effort than to light PA (Table 6).

Finally, the free time intervals that sometimes exist between the various school activities or between the different moments of the school day are also opportunities for children to play and exercise by the practice of PA. In this research, students from one of the centers studied (Center 2) have 60 min to have lunch and play before starting the afternoon session. Table 7 presents the records of PA, according to its intensity, performed during free times after lunch, recesses, and motor skills sessions. Table 8 presents the differences found in the different levels of physical activity between free times after eating, breaks and specific motor sessions.

The records obtained show a certain similarity to what happened during recess. During free play time after lunch, sedentary behavior had a mean duration of 43.3 ± 5.2 min, the time spent in MVPA was 11.7 ± 3.9 min and the mean total PA performed was 16.6 ± 5.2 min over a total duration of 60 min (Table 7).

According to Table 8, the levels of sedentary behavior are higher and similar in specific motor sessions and after lunch (43.3 ± 5.2 vs. 42.8 ± 5.2; *p* = 0.674). Lower levels of sedentary behavior are reached during recesses compared to motor sessions (39.3 ± 6.3 vs. 42.8 ± 5.2, *p* = 0.000) and after lunch (39.3 ± 6.3 vs. 43.3 ± 5.2; *p* = 0.000). In terms of MVPA, records were similar in specific motor sessions and after lunch (12.6 ± 4.5 vs. 11.7 ± 3.9; *p* = 0.312). The highest levels of MVPA were reached at recesses compared to motor sessions (14.9 ± 5.5 vs. 12.6 ± 4.5; *p* = 0.000) and to free time moments after lunch (14.9 ± 5.5 vs. 11.7 ± 3.9; *p* = 0.000). In terms of total PA or LVPA, the levels recorded in recesses are higher than those recorded in motor sessions (20.7 ± 6.3 vs. 17.2 ± 5.2; *p* = 0.000) and after lunch (20.7 ± 6.3 vs. 16.6 ± 5.2; *p* = 0.000). Therefore, we can affirm that the increase of the amount of recess time and free play moments during school hours implies an increase in the amount and intensity of PA performed by children, minimizing sedentary behaviors, thus confirming our third hypothesis.

## 4. Discussion

The purpose of this study was to explore the amount, intensity levels, and patterns of PA and sedentary behavior of preschool-age children throughout the school day and in its different times depending on the teaching methodology employed, determining their contribution to compliance with the recommendations established for this age group.

To reach this goal, we evaluated sedentary behavior and PA performed by children aged 4–5 years enrolled in 4 different centers in which Early Childhood Education is taught and in which different didactic methodologies are implemented. In all the centers, data were recorded using accelerometry. The total values of the school day were analyzed, as well as fractional values to examine children’s movement at different times such as recesses and other periods of free play, the specific motor sessions or the time dedicated to the teaching-learning academic content through different methodological approaches, which could be more or less active.

In addition, this research sought to study the characteristics present in these environments and during these moments to understand how they influence active or sedentary behaviors, finally proceeding to offer some guidelines or general proposals that can be used to promote movement integration in educational centers, thus increasing the PA performed by children during the school day and, therefore, reducing sedentary behavior in early childhood.

Overall, and based on the selected cut-off points [48], the results of our research show that most of the time children spend at school is dedicated to sedentary behaviors, with an average of approximately 80.7% of the total. Regarding the PA performed according to its intensity, we found that, on average, 6.5% of the day involves the implementation of light intensity activities, 6.7% is dedicated to moderate physical activity, and during 6.8% of the time spent at school, children engage in vigorous PA.

This pattern is consistent with other similar studies previously published, despite the heterogeneous nature of the schools in which the different investigations have been carried out. The levels of PA and sedentary behavior observed in Early Childhood Education centers in previous reports, in which PA levels in preschool children were studied during their stay in school for a period of one week, indicate that they spend most of their time sedentary, followed by light PA and MVPA [7,8,13,18,20,21,23,24,27,40,46,47].

Furthermore, with respect to the amount and intensity of PA performed, it has been observed that there are variations depending on the established schedule, the configuration of the classroom environment, teaching practices, and the free play opportunities offered throughout the school day, among others. In any case, children need to be encouraged to practice more PA on a daily basis, since what they do at school is not enough to reach an optimal level [8,19,21,23,28].

It should be recalled that there are reports published by various official bodies that consider that children under five years of age should perform a minimum of 180 min per day of PA of various types and of any intensity, of which at least 60 min should be of preferably aerobic PA and of moderate to vigorous intensity [35,36,37,38]. According to the data in Table 1, during the school day a child in these centers would meet, on average, 68% (40.8 min/day) of the recommendations for MVPA and in a 33.1% (59.6 min/day) those related to total PA.

As we can see, these figures do not reach the recommended minimums, especially those related to total PA. Although it is true that it is difficult to comply with these indications only from educational centers and during the time covered by the school day, it is possible to try to partially alleviate this problem by means of different strategies. We should not forget that schools are ideal settings to promote PA in early childhood due to the large number of hours that children spend in them, which maximizes their reach and guarantees access to the majority of the child population for prolonged and regular periods of time [2,16,19,20,26,27,31,40,41,42].

This is why identifying opportunities for PA in school is imperative to promote movement in the early childhood stage, especially if we use the regular classroom to combine PA with learning content through physically active academic lessons [7,20,27,28,40,42]. In fact, there are numerous interventions designed to increase PA in the classroom while continuing to work on the curriculum [5,7,16,18,20,26,27,40,42]. This has a double benefit, since not only does it get children to move more, but the type of movement they execute favors their cognitive functioning, promotes the development of executive functions and increases academic performance [3,4,11,18,26,32] without affecting the time allocated to the teaching-learning process [7,20,27,28,40].

Fortunately, teachers are increasingly trained and knowledgeable about the importance of PA and neurosciences, so they are continually creating interesting and meaningful experiences that combine movement with cognitively demanding practices in order for their students to students move, explore, act, manipulate and learn actively [1,12], improving both physical and cognitive skills [46].

### 4.1. Integrated Physical Activity (Physical Activity into the Classroom)

Although schools have been considered one of the best environments to implement interventions impregnated with PA, they seem to be one of the dominant settings for sedentary behavior in children, as we have observed in this study, and class time represents a significant sedentary period of the day. These results correspond to previous research reporting that children, when at school, spend too long periods of time sitting in the classroom [7,16,27,40].

Ironically, although the school environment is ideal for encouraging children to be physically active during the day, current curricula place almost all of their emphasis on academic content, especially literacy and numeracy, which has resulted in a detriment to PA [15,16,20]. To address this issue, we found that methods of movement integration into academic lessons can significantly increase the amount of PA children engage in during the school day.

Findings from the few previous studies identified focused on classroom interventions that integrate PA into academic content with preschoolers, demonstrate that movement integration significantly improves students’ PA levels during school hours without affecting the time or quality of academic instruction [3,6,16,20,28,40]. These results are consistent with those of our research, which shows that the children who spend more time sedentary (55.4 ± 2.4 min/h) and with lower levels of PA (AFMV = 2.7 ± 1.5 min/h; AFLV = 4.6 ± 2.4 min/h) are those involved in more traditional teaching methodologies (Table 3). In contrast, we have found that, in centers in which a mixed methodology is introduced and some movement is allowed, sedentarism decreases (52.5 ± 3.3 min/h) and PA increases (AFMV = 4.7 ± 2.4 min/h; AFLV = 7.4 ± 3. 3 min/h), being the center with a fully active methodology the one in which less sedentary behavior (46.6 ± 5.9 min/h) and better levels of PA (AFMV = 8.7 ± 4.3 min/h; AFLV = 13.1 ± 5.9 min/h) are observed.

In addition to these results, there is the added benefit that increased school-based PA leads to an improvement in cognitive skills and attitudes, academic behavior, classroom participation and engagement, and academic performance [11,15,27,31,41]. Thus, taking into account that cognitively engaging PA benefits executive function to a greater extent than that performed without a specific cognitive purpose [6], we can affirm that methodologies that integrate movement into academic content are one of the best ways to increase children’s PA levels while enhancing their cognitive development and learning [26,27].

However, to introduce movement integration practices in educational centers, it is necessary to take into account the characteristics of early childhood, mainly due to the immaturity of their psychosocial and cognitive capacities [25] and the different needs of the developing brain to provide, through activity, a pattern of varied stimulation of the environment that favors its own optimal development [33]. This is why, at these ages, children act impulsively, show more spontaneous activity patterns, can be easily distracted, have trouble waiting or sitting still for a prolonged period of time, and show little persistence in what they are doing as well as less interest in maintaining a single activity for prolonged periods of time [3,5,14,23,25,33].

In response to these distinctive features, positive and stimulating spaces such as those offered in Center 4, where young children are free to choose their own learning and allow for great flexibility and creativity, should be sought. Moreover, the use of play as a form of learning is consistent with respect for individualities and the different rates of growth and maturation [1,4].

### 4.2. Specific Motor Sessions

Psychomotricity is a discipline that tries to overcome body-mind dualism, promoting the execution of movement linked to the brain, the nervous system and cognition [32]. That is why specific motor sessions enhance motor development and are linked to children’s future PA habits, but they also provide a unique form of enrichment that strongly and positively affects children’s cognitive development [1,5,22], as well as empathy, tolerance, self-concept, self-confidence, intrinsic motivation, self-esteem and social skills [1,44].

Our study, in line with the results of previous research [1,32,44], shows the little time that is dedicated to specific motor sessions and the lack of spaces and facilities for the exclusive use of PA in preschoolers. Of the centers studied, only one provides for one hour of daily motor activity, another implements 3 sessions (with a 60-min class day longer than the rest of the centers), and the third only includes a weekly 60-min session. The last one, although it has a motor classroom that students can use whenever they want, does not put into practice specific and directed sessions.

However, research suggests that there are significant differences in PA levels between days with and without motor sessions, being those with this type of intervention where the greatest amount of movement is reached by children [19,32]. Subsequently, specific motor sessions are fundamental for the students’ level of PA. Table 5 shows that a 60-min motor session involves an average of 17.2 ± 5.2 min/h of PA, of which 12.6 ± 4.5 min/h are of MVPA.

It is important, therefore, to respect the time dedicated to these sessions, not replacing them by other activities that do not involve motor activity, or reducing its duration in order to transfer that time to other more sedentary activities. Moreover, as far as possible, it would be necessary to increase the weekly time allotted for motor sessions. This option was adopted by both Center 2 (with 3 h per week) and Center 3 (with 5 h per week), although it should be taken into account that the school day in Center 2 is longer, and this also implies an increase in sedentary activities. Therefore, we can affirm that the strategy of Center 2 achieves better overall results in relative terms.

Continuing along this line, we must also consider the need to make full use of the time provided for each session. The data analyzed reveal that, during a 60-min motor session, children spend a mean of 42.8 ± 5.2 min sedentary (Table 5) and only 17.2 ± 5.2 min of motor involvement were recorded. This corresponds to other studies in which it was found that, generally, a lot of time is lost due to reasons such as displacements, the organization of the material or the lack of concentration/distractions of the students during the activity [32].

When a one-hour motor skills session is planned, we must think that in that time it is necessary to move with the students to the space to be used, organize the material and the children, explain the activities to be carried out and, after finishing, pick up the material and go back to the regular classroom. That is why it would be necessary to have specific spaces and specialized and/or support teachers to help reduce these downtimes and make the most of the session possible.

On the other hand, the skills to be worked on in motor sessions do not always involve a displacement that translates into MVPA. It is necessary to include intentionally programmed opportunities and experiences for children to explore their motor possibilities, acquire general dynamic coordination and achieve greater control of movement [4], such as balance activities, climbing, quadruped, dragging, stretching, relaxation, body expression, etc.

Our study supports the hypothesis of other works in which the suitability of motor sessions is defended to promote motor and cognitive development together [4,13,18,44], since correctly applying motor skills in the classroom we could make the most of the physical and motor potential of the students, thus influencing the educational process.

### 4.3. Recess and Free Play

Although preschool-aged children are developmentally ready to participate in organized activities, it is also important that they are given the opportunity to engage in free, self-initiated motor play [25]. Studies have shown that different types of games, activities and exercises such as, for example, circle games or pretend play, promote not only an increase in children’s physical activity levels [33], but also an improvement in their executive functions [3].

For this reason, adopting measures such as increasing the number of recesses and/or taking advantage of other moments of free play (such as intervals between school activities or after lunch) is essential to increase children’s opportunities for PA. If we refer to our study, we find that even better results are obtained during breaks (Table 6) than in specific motor skills sessions (Table 5) in terms of sedentary behavior (39.3 ± 6.3 vs. 42.8 ± 5.2, *p* = 0.000), MVPA (14.9 ± 5.5 vs. 12.6 ± 4.5, *p* = 0.000) and total PA (20.7 ± 6.3 vs. 17.2 ± 5.2, *p* = 0.000). These results are in line with those of other researches that affirm that during recess children reach higher levels of PA [8,23], although it has also been found that the intensity of PA may depend on whether the play is totally free or structured [14].

It is undeniable that play is essential in early childhood as a means of development and learning. However, it is necessary to take into account that there must be an intention in it, since free play in early childhood education, as it takes place, ends up favoring sedentary behaviors more than MVPA. On the contrary, structured play sessions favor a significant increase in it provided they are organized around a pattern of intermittent and short-duration activities [14]. This suggests that it is not the free play sessions themselves that promote sedentary or active behaviors, but the way they are organized in a session.

The design of physically active interventions that integrate motor learning with academic content is emerging as a promising way to promote motor and cognitive development [3]. However, these proposals that combine motor and cognitive stimulation should be complemented by a wide variety of free play experiences, since they appear to amplify the benefits of motor-enriched educational programs [17]. 

Studies that analyzed the impact of physical activity during recess on academic performance found positive relationships suggesting that movement during recess is associated with improvements in attention, concentration and/or in-task classroom behavior [31]. More specifically, preschool children’s attention has been found to increase during classroom activities or other learning experiences when these are performed directly after playing at recess, so the authors conclude that play “rejuvenates” children’s brain functions [15].

Considering the above, we can affirm that it is most beneficial at these ages to introduce opportunities for free and active play along with academic curricular proposals, although more research is needed to explore the appropriate balance between free play, academic enrichment through movement integration and other organized activities [25].

### 4.4. Didactic Implications

Schools are an important setting for promoting PA in early childhood. However, school-based movement integration programs often face implementation challenges, so identifying the factors that influence this process is important to ensure the effectiveness of future interventions [8,13,16,17,41,42].

The characteristics of the teacher, the students and the environment, together with the management of resources and time, can influence movement integration into the school day. As for the professionals who teach the specific motor sessions in the centers studied, most are the teachers/tutors of the classroom group themselves, and only in Center 2 these sessions are taught by specialist teachers, which is consistent with other research [44]. It would be necessary for schools to incorporate specialized teachers in the same way as they do for teaching other disciplines such as, for example, foreign language. In this way, more would be made of the motor sessions and, in addition, they would be more effective in promoting cognitive and motor development of students.

Regarding movement integration into the academic content of the classroom, a possible suggestion to reduce the time children spend in sedentary behaviors is to raise teachers’ awareness of the negative consequences of sedentary time, along with training teachers to design and implement physically active lessons that progressively replace sedentary activities [13,21]. Additionally, the amount of real and perceived time available to the teacher and the need to meet curriculum objectives can also affect their ability to plan and deliver movement integration [16,21,27,41]. At this point, it is important to note that having the support of the school principal, valuing and prioritizing movement integration within the school, is essential for the success of PA-based interventions [16,27,28,41].

Beyond teacher’s characteristics and cognitive attributes, the children themselves, their disposition and their individual characteristics also play an important role in whether movement integration is incorporated into the classroom, since if students do not have a positive attitude towards PA, movement integration experiences will hardly be successful [27,41,42]. That is why we must plan the space, materials, and time correctly so that they generate learning while offering children attractive and interesting proposals in which play, experimentation and practical activity constitute the main vehicle for teaching the contents [1].

There are numerous didactic implications that can be drawn from this study for professional practice in preschoolers. Data reveal that, while achieving a high level of MVPA requires the use of free play and specially targeted structured practice or motor sessions, reducing overall levels of sedentary lifestyles and increasing total PA requires the use of active methodologies that allow and encourage movement during the school day.

In our research, two of them have been evaluated: the methodology based on learning corners within the classroom and that of open concept classrooms with shared spaces that allow children to move around the different classrooms, the playground and other areas. Although both methodologies contribute to reducing sedentary behaviors and increasing the total PA carried out during the school day, it is in the last one that the best results were obtained.

For this reason, and in agreement with other authors [1,19], we consider that the best way to develop academic learning that also promotes the regular practice of PA is the creation of open classrooms. The reason is that they can be used to work on different contents and skills previously planned by the teacher, and include the educational habilitation of other indoor and outdoor spaces of the center, which should be accessible at any time and not only at recess.

In relation to specific motor sessions, one of the biggest problems detected in our study is that the centers do not have a specific space for them, since sports facilities are usually reserved for children in higher educational stages and preschoolers usually end up using some unspecific space, generally outdoors. While it is interesting to note that outdoor activity stimulates variation in children’s movements and provides a natural terrain for cognitive stimulation [18], centers that use indoor spaces for gross motor activities (rather than relying solely on the possibility of using those outdoors) accumulate less time of sedentary behaviors [8,14].

It is also important that in these sessions the deliberate practice of PA can be reconciled with forms of free play developed in previously prepared environments to work on specific skills, always with the guidance and accompaniment of the teacher. It has been shown that structuring motor sessions in the form of deliberately prepared play, emphasizing enjoyment and participation in a variety of activities and focusing on the acquisition of fundamental motor skills through developmentally appropriate tasks, can have a positive influence on physical, cognitive and social development in early childhood [1,18,20].

Finally, it is also necessary to schedule times in the day that provide free play periods for children, especially before academic content learning activities take place. This is because free play favors that, once children return to class, they show greater task-engagement and achieve greater learning [23]. Therefore, we agree with other studies that conclude that the ideal would be to schedule two free-play periods throughout the school day, each placed before a learning time [15,23].

However, we should not forget that totally free play ends up favoring sedentary behavior, so that children during free play may be more active at the beginning, but they do not maintain the same level of activity throughout the session [8,14]. Here, the role of teachers is essential in facilitating children to maintain greater participation in play for longer periods, especially thanks to the instructions and motivation they can offer.

Teachers can create play spaces and draw playground markings, as well as offer materials that are known to inspire increased movement, such as portable play equipment (balls, hoops, cones, etc.) and natural elements (water, grass, sand, mud, etc.). They can also interact with children in ways that promote higher levels of PA, such as initiating active play and movement activities, offering new equipment, and inviting sedentary children to join their peers in active play [8,15].

To sum up, to ensure a successful movement integration into the academic content of the classroom, it is necessary to train teachers in new and more active methodologies based on physically active lessons. Nevertheless, it is also necessary to provide them with time to design new programs and to prepare the classroom and materials and with the resources that movement integration practices require.

For their part, teachers have to ensure that movement integration activities are attractive to the students, and therefore they should carefully plan the organization of the space and the materials so that they allow a type of learning that is both playful and meaningful. In this sense, the best methodology seems to be that based on open concept classrooms, since it is in it that the best results are obtained promoting PA.

Moreover, it is necessary to have indoor facilities dedicated exclusively to specific motor sessions, with the space, equipment and material necessary to be able to work together all the physical and cognitive areas of children development. In addition, it is essential to merge in these sessions the deliberate practice of PA to work on specific skills with some moments of free play. For all this, it would be helpful to have specialized teachers in early childhood PA in schools in order to maximize the potential of these sessions.

Lastly, children need free play time during the school day to achieve greater task-engagement and learning. For this, it might be ideal to schedule two recesses, preferably of a kind of deliberate free play that can be achieved by different means. For example, it is possible to create play spaces with natural elements such as water, grass, sand or mud or drawing marks in playgrounds. Teachers can also motivate children, interact with them, initiate play activities and teaching them new games, in addition to providing play materials such as balls, hoops, cones, ropes, slides, etc.

As we can see, the results of the present study have numerous important conceptual and practical implications that can provide further insight into the value of movement integration, as well as the factors and resources needed for its successful implementation. Despite the evidence that movement integration into the school day leads to an increase in PA in children, it is also clear that many factors affect the degree to which teachers use it, being very uneven the amount of PA taking place in different schools. The results of this research may shed light on the key factors that facilitate the initiation and long-term maintenance of movement integration along with other practices that promote children’s exercise.

### 4.5. Study Limitations and Future Research

This study has limitations that need to be addressed. First, the use of accelerometers had numerous advantages, but also some disadvantages that should be taken into account. On the one hand, its use minimized reporting bias, provided an objective measure of children’s sedentary behavior and PA at different intensities, and increased the validity of the external results. However, accelerometers underestimate the intensity of certain PA that, although intense, do not necessarily involve displacements or accelerations (so are not captured by accelerometers). Nor can they accurately measure the additional energy expenditure of carrying a load while movement occurs, at the same time that they may overestimate other activities that are of relatively low intensity, such as playing on a swing. This has been observed in other studies, which specified that the type of activity performed (e.g., running or jumping versus climbing) influenced the measured activity levels [8,23,46].

Another limitation is that the physical characteristics of the spaces (such as their dimensions, equipment, rules of use, etc.) were not recorded, nor the specific materials or activities offered in the centers studied. Teachers’ interactions with the students during the different moments of the school day analyzed were not examined either. The findings of our study could be affected by differences in the behavior and role of teachers in the time dedicated to teaching academic content, at recess or in specific motor sessions. In this regard, research on adults’ interactions with preschool children at recess and how they later impact on children’s classroom behavior and learning is of great interest [15].

In the future, in order to continue, complete and expand this research, it would be interesting to include in the study a greater number of schools located in a larger geographical area, so that we can have more data from which to draw conclusions and didactic implications for the promotion of PA. 

It is also possible to convert the research into a longitudinal design in which different types of interventions would be implemented in order to test their effects on increasing the amount and intensity of PA performed by students, as well as on reducing sedentary behaviors. Similarly, some type of hierarchical linear modeling approach could be applied in order to capture the effects of different educational practices on the PA levels of preschool children.

## 5. Conclusions

This research was designed to address gaps in our understanding of the implications for the quantity and intensity of PA performed during physically active academic lessons in preschool settings. The lack of early childhood interventions that integrate PA with academic content in a meaningful way highlights the need for work such as this.

We should not forget that the classroom is where students spend most of their time and this provides a viable venue for interventions designed to increase PA. However, although the potential of these interventions (especially those implemented long term) to improve both PA and academic performance has been demonstrated, given that movement integration practices are still relatively new, research related to these processes is important to enable the development of more effective future interventions.

Understanding, promoting, and evaluating PA in very young children is challenging, but it is necessary to think beyond the framework of intensity, duration and frequency of activity and ask what types are most beneficial to children’s overall and holistic development. Evidence now consistently supports that not all forms of exercise influence cognition equally, with those requiring complex, controlled, and adaptive cognition and movement having the greatest impact on executive functions.

Therefore, if we want to increase children’s PA during the school day, thus contributing to reaching the official recommendations, it is necessary to take some measures based on three specific lines of action. First of all, it is essential to promote teaching strategies and styles that increase students’ activity levels, such as active learning methodologies that integrate academic content with motor activity, and in which students have to move, participate dynamically and become involved motorly by the creation of open classrooms, specific areas for motor exercise and large play spaces.

Secondly, not only the number of specific motor sessions should be increased, but also the time of motor engagement that takes place in each of them. To achieve this, some measures can be implemented, including the prior preparation of spaces and materials, so that time does not have to be repeatedly wasted placing and collecting the material, the selection of tasks that require a brief explanation and a simultaneous and intense exercise of the students, and the incorporation into schools of specialized teachers.

In the third place, play, particularly active and free play, should be reestablished in children’s lives without subordinating it to other activities such as breakfast or the completion of other tasks. It is necessary for schools to increase the number of recesses and adjust their duration so that they are used as much as possible, avoiding sedentary behaviors due to exhaustion or lack of motivation. To this end, it can be beneficial to induce children to engage in PA by offering them a variety of activities, materials or equipment.

One of our purposes with this research is to make these data available to the educational community to provide an incentive for the dissemination and implementation of more active pedagogies by teachers, helping to guide the design of future school interventions. Ultimately, what children do in the classroom is largely up to the teacher and, consequently, making a change in the classroom is ultimately a personal and individual decision of the teachers. Therefore, we seek to encourage teachers to integrate PA into academic lessons by presenting them with affordable options that can fit their schedule, curriculum, and possibilities.

## Figures and Tables

**Table 1:** Daily PA in minutes and percentage performed by students during school hours.

	Center 1 (N = 46)			Center 2 * (N = 39)			Center 3 (N = 47)			Center 4 (N = 24)		
	*M*	*SD*	*%*	*M*	*SD*	*%*	*M*	*SD*	*%*	*M*	*SD*	*%*
Sedentary	247.3 ± 11.6		82.4	280.0 ± 11.5		77.7	240.2 ± 12.3		80.6	237.5 ± 11.0		79.1
Light	17.8 ± 3.2		5.9	19.0 ± 3.0		5.3	17.9 ± 3.4		6.0	20.7 ± 2.6		6.9
Moderate	17.7 ± 4.1		5.9	20.6 ± 3.7		5.7	20.0 ± 4.1		6.7	21.3 ± 3.5		7.1
Vigorous	17.2 ± 5.2		5.7	23.9 ± 5.9		6.6	21.8 ± 6.4		7.3	20.6 ± 5.9		6.9
MVPA	34.9 ± 8.9		11.6	44.6 ± 8.1		12.3	41.9 ± 9.6		14.0	41.8 ± 9.0		13.9
LVPA	52.7 ± 11.5		17.5	63.5 ± 10.3		17.6	59.7 ± 12.3		19.9	62.5 ± 11.0		20.8

Note 1: PA= Physical Activity; N = Total number of cases; M = Mean; SD = Standard Deviation; MVPA = Moderate to vigorous physical activity; LVPA = Light to vigorous physical activity. Note 2: * Methodology with 360 min of daily school hours (300 min for the other methodologies).

**Table 2:** Differences found between PA performed and the different intensities among centers.

Centers			Sedentary	Light	Moderate	Vigorous	MVPA	LVPA
1 (N = 46)	2	p Z	0.000 * −7.734	0.049 −1.971	0.002 * −3.087	0.000 * −4.912	0.000 * −4.815	0.000 * −4.383
	3	p Z	0.004 * −2.874	0.866 −0.169	0.009 * −2.613	0.000 * −3.742	0.000 * −3.604	0.004 * −2.874
	4	p Z	0.001 * −3.205	0.000 * −3.588	0.001 * −3.359	0.015 * −2.437	0.003 * −2.982	0.001 * −3.205
2 (N = 39)	1	p Z	0.000 * −7.734	0.049 −1.971	0.002 * −3.087	0.000 * −4.912	0.000 * −4.815	0.000 * −4.383
	3	p Z	0.000 * −7.898	0.074 −1.787	0.353 −0.928	0.056 −1.913	0.057 −1.904	0.060 −1.878
	4	p Z	0.000 * −6.623	0.016 −2.420	0.533 −0.622	0.015 −2.434	0.179 −1.344	0.640 −0.467
3 (N = 47)	1	p Z	0.004 * −2.874	0.866 −0.169	0.009 * −2.613	0.000 * −3.742	0.000 * −3.604	0.004 * −2.874
	2	p Z	0.000 * −7.898	0.074 −1.787	0.353 −0.928	0.056 −1.913	0.057 −1.904	0.060 −1.878
	4	p Z	0.313 −1.009	0.000 * −3.658	0.135 −1.495	0.415 −0.814	0.913 −0.109	0.313 −1.008
4 (N = 24)	1	p Z	0.001 * −3.205	0.000 * −3.588	0.001 * −3.359	0.015 −2.437	0.003 * −2.982	0.001 * −3.205
	2	p Z	0.000 * −6.623	0.016 * −2.420	0.533 −0.622	0.015 −2.434	0.179 −1.344	0.640 −0.467
	3	p Z	0.313 −1.009	0.000 * −3.588	0.135 −1.495	0.415 −0.814	0.913 −0.109	0.313 −1.008

Note 1: N = Frecuency; MVPA = Moderate to Vigorous Physical Activity; LVPA = Light to Vigorous Physical Activity; p = statistical significance; Z = normal approximation. Note 2: Mann-Whitney U test to compare medians between centers. Bonferroni correction * *p* ≤ 0.012.

**Table 3:** Recording in minutes of performed PA according to teaching methodology.

	Traditional (HT = 276)			Learning Corners (HT = 169)			Open Classroom Concept (HT = 316)		
	*M*	*SD*	*%*	*M*	*SD*	*%*	*M*	*SD*	*%*
Sedentary	55.4 ± 2.4		92.3	52.5 ± 3.3		87.5	46.6 ± 5.9		77.6
Light	1.9 ± 0.9		3.1	2.7 ± 1.0		4.5	4.5 ± 1.5		7.5
Moderate	1.7 ± 0.9		2.8	2.5 ± 1.2		4.2	4.6 ± 1.9		7.6
Vigorous	1.1 ± 0.7		1.8	2.2 ± 1.4		3.7	4.1 ± 2.6		6.8
MVPA	2.7 ± 1.5		4.5	4.7 ± 2.4		7.8	8.7 ± 4.3		14.5
LVPA	4.6 ± 2.4		7.6	7.4 ± 3.3		12.3	13.1 ± 5.9		21.8

Note: HT = Total number of hours recorded; M = Mean; SD = Standard Deviation; MVPA = Moderate to vigorous physical activity; LVPA = Light to vigorous physical activity.

**Table 4:** Differences found among teaching methodologies and PA performed with different intensities.

	Teaching Methodology			Sedentary	Light	Moderate	Vigorous	MVPA	LVPA
TH = 276	Tra	LC OCC	p Z	0.000 * −9.005	0.000 * −8.119	0.000 * −7.609	0.000 * −9.868	0.000 * −8.972	0.000 * −9.005
			p Z	0.000 * −18.590	0.000 * −18.297	0.000 * −17.889	0.000 * −17.236	0.000 * −18.026	0.000 −18.433
TH = 169	LC	Tra OCC	p Z	0.000 * −9.005	0.000 * −8.119	0.000 * −7.609	0.000 * −9.868	0.000 * −8.972	0.000 * −9.005
			p Z	0.000 * −11.755	0.000* −12.303	0.000* −11.769	0.000* −9.254	0.000* −10.831	0.000 * −11.577
TH = 316	OCC	Tra LC	p Z	0.000 * −18.590	0.000 * −18.297	0.000 * −17.889	0.000 * −17.236	0.000 * −18.026	0.000 * −18.433
			p Z	0.000 * −11.755	0.000 * −12.303	0.000 * −11.769	0.000 * −9.254	0.000 * −10.831	0.000 * −18.433

Note 1: TH = Number of total hours recorded; MVPA = Moderate to Vigorous Physical Activity; LVPA = Light to Vigorous Physical Activity; Tra = Traditional methodology; LC = Learning Corners; OCC = Open Concept Classroom. Note 2: Mann-Whitney U test to compare medians between centers. Bonferroni correction * *p* ≤ 0.016.

**Table 5:** Minutes recorded of PA performed in the specific motor sessions.

	Center 1 (SS = 36)		Center 2 (SS = 84)		Center 3 (SS = 259)		Total (SS = 379)	
	*M*	*SD*	*M*	*SD*	*M*	*SD*	*M*	*SD*
Sedentary	47.4 ± 4.2		39.1 ± 4.4		43.3 ± 5.0		42.8 ± 5.2	
Light	4.2 ± 1.2		5.2 ± 1.0		4.4 ± 1.1		4.6 ± 1.1	
Moderate	4.0 ± 1.4		6.5 ± 1.7		5.5 ± 1.5		5.6 ± 1.7	
Vigorous	4.3 ± 2.0		9.2 ± 3.1		6.7 ± 2.9		7.1 ± 3.2	
MVPA	8.4 ± 3.1		15.7 ± 4.0		12.2 ± 4.2		12.6 ± 4.5	
LVPA	12.6 ± 4.2		20.1 ± 4.4		16.7 ± 4.9		17.2 ± 5.2	

Nota 1: SS = Total number of 1-h sessions recorded; M = Mean; SD = Standard Deviation; MVPA = Moderate to vigorous physical activity; LVPA = Light to vigorous physical activity. Note 2: Center 4 does not carry out any specific motor session.

**Table 6:** PA performed in the different centers during recesses *.

	Center 1 (TR = 169)		Center 2 (TR = 138)		Center 3 (TR = 192)		Center 4 (TR = 102)		Total (TR = 601)	
	*M*	*SD*	*M*	*SD*	*M*	*SD*	*M*	*SD*	*M*	*SD*
Sedentary	42.1 ± 4.6		39.1 ± 5.8		35.5 ± 6.6		42.0 ± 5.1		39.3 ± 6.3	
Light	5.6 ± 1.4		5.4 ± 1.3		6.2 ± 1.3		5.7 ± 1.4		5.8 ± 1.4	
Moderate	6.0 ± 1.9		6.5 ± 1.9		7.8 ± 2.1		6.1 ± 2.0		6.7 ± 2.1	
Vigorous	6.3 ± 2.4		9.0 ± 3.5		10.4 ± 4.6		6.1 ± 2.4		8.2 ± 3.9	
MVPA	12.3 ± 3.8		15.6 ± 4.9		18.2 ± 6.0		12.2 ± 4.1		14.9 ± 5.5	
LVPA	17.9 ± 4.6		20.9 ± 5.8		24.5 ± 6.6		18.0 ± 5.1		20.7 ± 6.3	

Note 1: TR = Total Hours of Recesses recorded; M = Mean; SD = Standard Deviation; MVPA = Moderate to vigorous physical activity; LVPA = Light to vigorous physical activity. Note 2: * 1-h interval including time for breakfast.

**Table 7:** PA performed during free time after lunch, recess and specific motor sessions.

	After Lunch (AL = 53)		Recesses (RH = 601)		Motor Sessions (MS = 379)	
	*M*	*SD*	*M*	*SD*	*M*	*SD*
Sedentary	43.3 ± 5.2		39.3 ± 6.3		42.8 ± 5.2	
Light	4.9 ± 1.7		5.8 ± 1.4		4.6 ± 1.1	
Moderate	5.4 ± 1.8		6.7 ± 2.1		5.6 ± 1.7	
Vigorous	6.3 ± 2.6		8.2 ± 3.9		7.1 ± 3.2	
MVPA	11.7 ± 3.9		14,9 ± 5.5		12.6 ± 4.5	
LVPA	16.6 ± 5.2		20.7 ± 6.3		17.2 ± 5.2	

Note 1: AL = Total number of After Lunch records; RH = Total number of Recess Hours recorded; MS = Total number of Motor Sessions recorded; M = Mean; SD = Standard Deviation; MVPA = Moderate to Vigorous Physical Activity; LVPA = Light to Vigorous Physical Activity.

**Table 8:** Differences between PA intensities performed in the free time after lunch, recesses and specific motor sessions.

	Time of School Day			Sedentary	Light	Moderate	Vigorous	MVPA	LVPA
TH = 276	AL	RC MS	p Z	0.000 * −4.416	0.000 * −3.566	0.000 * −4.0840	0.001 * −3.175	0.000 * −4.105	0.000 * −4.416
			p Z	0.674 −0.421	0.024 −2.248	0.857 −0.180	0.241 −1.173	0.312 −1.011	0.674 −0.421
TH = 169	RC	AL MS	p Z	0.000 * −4.416	0.000 * −3.566	0.000 * −4.0840	0.001 * −3.175	0.000 * −4.105	0.000 * −4.416
			p Z	0.000 * −8.550	0.000 * −13.253	0.000 * −8.587	0.000 * −4.249	0.000 * −6.455	0.000 * −8.550
TH = 316	MS	AL RC	p Z	0.674 −0.421	0.024 −2.248	0.857 −0.180	0.241 −1.173	0.312 −1.011	0.674 −0.421
			p Z	0.000 * −8.550	0.000 * −13.253	0.000 * −8.587	0.000 * −4.249	0.000 * −6.455	0.000 * −8.550

Note 1: TH = Number of total hours recorded; MVPA = Moderate to Vigorous Physical Activity; LVPA = Light to Vigorous Physical Activity; AL = After Lunch; RC = Recesses; MS = Motor Sessions. Note 2: Mann-Whitney U test to compare medians between centers. Bonferroni correction * p ≤ 0.016.

## Data Availability

The individual data of the participants are not publicly available, as specified in the original approval by the Ethics Committee of research with human beings of the Universidad de Málaga, Spain (protocol code 114-2020-H; approved on 26 February 2021) and in the informed consent from the participants and their respective legal guardians.
